# Computational and experimental methods for classifying variants of unknown clinical significance

**DOI:** 10.1101/mcs.a006196

**Published:** 2022-04

**Authors:** Malte Spielmann, Martin Kircher

**Affiliations:** 1 Institute of Human Genetics, University of Lübeck, 23562 Lübeck, Germany;; 2 Institute of Human Genetics, Christian-Albrechts-Universität, 24105 Kiel, Germany;; 3 Human Molecular Genomics Group, Max Planck Institute for Molecular Genetics, 14195 Berlin, Germany;; 4 DZHK (German Centre for Cardiovascular Research), partner site Hamburg/Lübeck/Kiel, 23562 Lübeck, Germany;; 5 Berlin Institute of Health at Charité—Universitätsmedizin Berlin, 10117 Berlin, Germany;; 6 DZHK (German Centre for Cardiovascular Research), partner site Berlin, 10115 Berlin, Germany

## Abstract

The increase in sequencing capacity, reduction in costs, and national and international coordinated efforts have led to the widespread introduction of next-generation sequencing (NGS) technologies in patient care. More generally, human genetics and genomic medicine are gaining importance for more and more patients. Some communities are already discussing the prospect of sequencing each individual's genome at time of birth. Together with digital health records, this shall enable individualized treatments and preventive measures, so-called precision medicine. A central step in this process is the identification of disease causal mutations or variant combinations that make us more susceptible for diseases. Although various technological advances have improved the identification of genetic alterations, the interpretation and ranking of the identified variants remains a major challenge. Based on our knowledge of molecular processes or previously identified disease variants, we can identify potentially functional genetic variants and, using different lines of evidence, we are sometimes able to demonstrate their pathogenicity directly. However, the vast majority of variants are classified as variants of uncertain clinical significance (VUSs) with not enough experimental evidence to determine their pathogenicity. In these cases, computational methods may be used to improve the prioritization and an increasing toolbox of experimental methods is emerging that can be used to assay the molecular effects of VUSs. Here, we discuss how computational and experimental methods can be used to create catalogs of variant effects for a variety of molecular and cellular phenotypes. We discuss the prospects of integrating large-scale functional data with machine learning and clinical knowledge for the development of accurate pathogenicity predictions for clinical applications.

## FROM THE FIRST DRAFT GENOME TO FUNCTIONAL ANNOTATIONS

One central question in human genetics is the understanding of how genomic variation affects genome function and influences phenotypes. The Human Genome Project was the foundation for many breakthroughs in our understanding of human genomic variation and the role it plays in health and disease (Gibbs 2020). Joint efforts of a broad community of biomedical researchers and two decades of large-scale projects including ENCODE (ENCODE Project Consortium et al. 2007; The ENCODE Project Consortium 2012; Moore et al. 2020), IHEC (Stunnenberg et al. 2016), NIH RoadMap Epigenomics (Satterlee et al. 2019), or FANTOM (Carninci et al. 2005; Abugessaisa et al. 2021) achieved tremendous progress in mapping the “functional” genome as various annotations layers to the reference genome. Other efforts like the International HapMap Project (International HapMap Consortium 2005), 1000 Genomes Project (1000 Genomes Project Consortium et al. 2015), UK10K (Walter et al. 2015), The Simons Genome Diversity Project (Mallick et al. 2016), and the Genome Aggregation Database (gnomAD) (Karczewski et al. 2020), as well as studies of structural variants (Sudmant et al. 2015; Collins et al. 2020; Ebert et al. 2021), helped cataloging human genetic variation. Efforts of large-scale cohorts and detailed phenotypic characterization are the basis for better functional mapping and gene association studies (Manolio and Collins 2009; Li et al. 2017; Bycroft et al. 2018). Most recently, the Telomere-to-Telomere (T2T) consortium is releasing full-length chromosomal sequences (Logsdon et al. 2021), enabling complete catalogs of human genetic sequence (Aganezov et al. 2021).

Although all this resulted in an immense knowledge gain, it also shows that in addition to the static mapping of genomic function and variation, over the next decade, we need to apply an efficient toolbox to engineer genomic alterations and to read out their functional effects in biological systems. In a recent effort, for example, the National Human Genome Research Institute (NHGRI) Impact of Genomic Variation on Function (IGVF) Consortium was established to utilize available and develop improved approaches to evaluate the function and phenotypic outcomes of genomic variation (National Human Genome Research Institute (NHGRI) 2021).

Meanwhile, genomic analyses of populations or individuals to identify disease-associated genomic variants are becoming routine, and clinicians, genetic counselors, and researchers are in need to classify an ever-increasing number of variants of uncertain significance (VUSs) between benign and pathogenic. Diagnostic assays such as newborn screening, exome and panel sequencing to diagnose Mendelian disorders or cancer, and noninvasive prenatal diagnosis (NIPT) tests are among the first high-throughput technology applications to have entered the clinic. With further decreasing costs, whole-genome sequencing will be the default genetic assay within the next years. Three to four million short sequence variants (i.e., single-nucleotide variants [SNVs], multibase substitutions, and insertion/deletion [indel] changes below 50 bp) as well as about 15,000 structural variants (SVs) are identified from an individual's genome (Acuna-Hidalgo et al. 2016; Ebert et al. 2021). Because of sheer numbers, the consideration of variant combinations on a genome-wide scale is intractable and variants need to be efficiently filtered (e.g., by using related individuals and their affected/unaffected status).

Already available variant catalogs and allele frequency thresholds provide a powerful tool for reducing the number of considered variants (Eilbeck et al. 2017; Shah et al. 2018). However, establishing causal relationships between variants and disease risk is still hampered by a lack of mechanistic understanding for interpreting filtered variants. Similarly, understanding the clinical relevance of variants is hindered by the overwhelming and ever-growing number of VUSs. Here, we discuss the various strategies including computational variant effect prediction, experimental assays, data sharing, and data integration developed for addressing the challenges posed by VUSs.

## PUBLIC RESOURCES AND THEIR APPLICATION IN THE IDENTIFICATION OF DISEASE CAUSAL VARIANTS

Largely driven by the availability of a reference genome and the development of cheaper sequencing methods (Kircher and Kelso 2010; Goodwin et al. 2016; Shendure et al. 2017; Gibbs 2020), the identification of disease causal variants and disease genes has seen a rapid advance over the last 15 years (Bamshad et al. 2019). The development of targeted sequencing using sequence capture and targeted amplification approaches (Hodges et al. 2007; Ng et al. 2009; Turner et al. 2009; Briggs 2011) has led to widely used, optimized, and commercialized laboratory kits to obtain high-quality sequence data of the exonic part of the genome (i.e., exome sequencing [ES]) or other clinically relevant sequences (e.g., panel sequencing). Reductions in sequencing costs have enabled a wider inclusion of sequencing of unaffected relatives (e.g., parent–child trios, quads including unaffected siblings, up to larger pedigrees), allowing for more effective identification of disease causal variants. The large number of studies and a broader inclusion of relatives revealed de novo variants and genetic mosaicism as a major source of Mendelian-type rare diseases (Campbell et al. 2015; Acuna-Hidalgo et al. 2016) and stimulated a transition from “phenotype-driven” to “genotype-driven” syndrome delineation in Mendelian disorders (Bamshad et al. 2019).

With observations like that, the field learned to appreciate that a dogmatic use of terminology has its limitations. Specifically, the identification of damaging variants and variants that alter molecular function is only a first of several steps toward the reporting of pathogenic variants—that is, the presence of a variant that is (potentially) causing disease (Eilbeck et al. 2017). We learned to appreciate that dosage effects (i.e., levels of gene expression) and haploinsufficiency, which we previously simplified in concepts like recessive and dominant disease, are measured on a continuous scale of gene expression and can be dependent on certain cell types as well as developmental programs. Rather than trying to maintain a black-and-white distinction between pathogenic and benign by introducing concepts of penetrance and variable expressivity, we need to incorporate the concept of health burden and the contribution of many genetic and environmental factors in the study of disease (Shendure and Akey 2015; Wang et al. 2021).

Public databases play a central role to strengthen our understanding of genomic variation in the context of disease, they help to facilitate the exchange of genetic variation and phenotype information. The database Online Mendelian Inheritance in Man (OMIM) aims to be a comprehensive and authoritative compendium of human genes and genetic phenotypes (Amberger et al. 2019). The database was initiated by Dr. Victor A. McKusick as a catalog of Mendelian traits and disorders and first published in 1966. The online version, OMIM, was created in 1985. At the beginning of 2006, OMIM cataloged approximately 15,800 entries. By the end of 2021, this number had increased to more than 26,000. In other words, the database grew by 66% in just the last quarter of its existence. Although this is already impressive, a recent study suggests that the number of delineated syndromes will continue to increase at high rates (Bamshad et al. 2019). Between phenotype ontologies (Köhler et al. 2019) and “genotype-driven” syndrome delineation, new concepts seem required to catalog genetic variant effects.

Another major step toward understanding normal genetic variation was the establishment of large variant databases. Even though the 1000 Genomes Project (1000 Genomes Project Consortium et al. 2015) and a number of other studies were instrumental in cataloging human genetic diversity, their allele frequency resolution was still insufficient for rare disease analyses. When the first large-scale exome studies came about, the NHLBI GO Exome Sequencing Project (ESP) set out to discover novel genes and mechanisms contributing to heart, lung, and blood disorders. The release of allele frequency information from the more than 6500 unrelated ESP individuals of African–American or European–American descent gave a first glimpse of the power of such data in the summer of 2012 (Tennessen et al. 2012; Fu et al. 2013). This idea motivated the Exome Aggregation Consortium (Lek et al. 2016) and later gnomAD (Karczewski et al. 2020), with the goal of aggregating and harmonizing both exome and genome sequencing data from a wide variety of large-scale sequencing projects and making summary data available for the wider scientific community. Especially the gnomAD database, with its intuitive web interface and additional variant and gene annotations, is currently being used in clinical laboratories around the world to filter for rare disease causing variants as the cause of Mendelian disorders.

In 2013, the American College of Medical Genetics and Genomics (ACMG) developed guidelines for the interpretation of sequence variants for clinical laboratories (Rehm et al. 2013; Richards et al. 2015). These recommendations currently represent the gold standard for tests used in clinical laboratories, including genotyping, single genes, panels, exomes, and genomes. The guidelines recommend the use of specific standard terminology—“pathogenic,” “likely pathogenic,” “uncertain significance,” “likely benign,” and “benign”—to describe variants identified in genes that cause Mendelian disorders. Overall, these diagnostic guidelines are quite “strict” because misidentifying a variant as pathogenic could have very severe consequences—for example, termination of a healthy fetus or an unnecessary surgical or invasive procedure.

**Figure 1.**
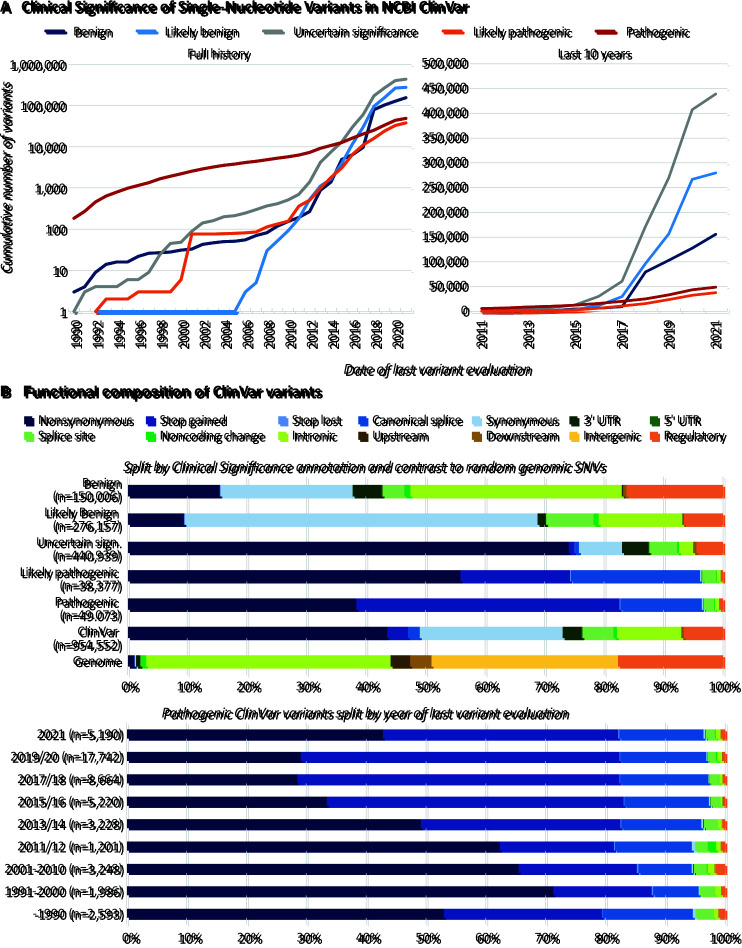
Rising numbers of variants of uncertain significance (VUSs) and the functional composition of ClinVar variants. (*A*) The number of variants with clinical assertions in NCBI ClinVar (Landrum and Kattman 2018) increased considerably in the last decade, but VUSs represent the largest class. As of its January 2022 release, ClinVar reports on more than 1.1 million variants. Shown is the number of GRCh38 single-nucleotide variants (SNVs) reported by their last date of variant evaluation (as a proxy for how long the variant has been known as the database was only established in 2013) and the assigned clinical significance (ClinSig) from 1990 to 2020 (*left*, logarithmic scale) and the last 10 years (*right*, linear scale). Entries without a date were excluded and only the nine most frequently used ClinSig values retained. In the remaining 961,829 entries, the nine levels were further simplified to five categories by assigning “Pathogenic/Likely pathogenic” (*n* = 7821) with “Likely pathogenic” (*n* = 32,421), “Benign/Likely benign” (*n* = 24,476) with “Likely benign” (*n* = 258,515) as well as “Conflicting interpretations of pathogenicity” (*n* = 51,298) and “not provided” (*n* = 8721) together with “Uncertain significance” (*n* = 392,706). By 2015, the number of VUSs exceeded the number of reported “Pathogenic” variants. (*B*) Annotated variant consequences for variants in ClinVar versus potential genomic SNVs highlight clear ascertainment effects. Using SNVs from panel *A*, we retrieved variant consequence annotation as reported feature by the Combined Annotation Dependent Depletion (CADD) v1.6 tool (Rentzsch et al. 2021) and 250,000 potential SNVs from the whole-genome CADD annotation file as representation of the genomic background. The *top* panel shows ClinVar variants by their clinical assertion, highlighting coding variants as dominant variant classes and upstream, downstream, and intergenic variants being generally underrepresented. Between clinical assertions, functional class representation follows classical observations of most severe effects for nonsense (stop gain) and missense (nonsynonymous amino acid exchanges) variants. The *bottom* panel highlights that also in recent years pathogenic variants do not show a substantial increase in the representation of noncoding variants.

Also in 2013 and out of clinical need, ClinVar was established as a public database for clinical laboratories, researchers, expert panels, and others to share their interpretations of variants with their evidence. The National Center for Biotechnology Information (NCBI) database aggregates information about genomic variation and its relationship to human health, specifically their clinical assertions (Landrum and Kattman 2018). Looking at this database, the recent progress in identifying disease causal variants is even more impressive with the number of “Pathogenic” variants tripling within the last 7 years. As of its January 2022 release, the database reports on more than 1.1 million variants, with more than 400,000 being annotated as VUS or “Uncertain significance.” For several years now, these clinically uncertain and not actionable variants represent a majority of annotated variants (Fig. 1A). Although this is already clear evidence that we are considerably lacking behind in variant characterization, gnomAD reports on more than 759 million short-sequence variants observed across more than 76,000 genomes in its v3.1 release.

Despite the overall progress in identifying disease causal variants and continued reports of new pathogenic variants and disease genes, over the last years, the highest reported diagnostic yields from exome and genome sequencing do not exceed 40%–60% depending on disease cohort (Lionel et al. 2018; Fung et al. 2020; 100,000 Genomes Project Pilot Investigators et al. 2021; Stranneheim et al. 2021). One widely discussed potential reason might be the persisting focus on coding sequence, short sequence variants, and our limited understanding of noncoding molecular processes to assess the potential effects of the vast majority of genomic variants.

## CODING AND NONCODING SEQUENCES

In the past, a major focus in identifying disease causal variants has been on coding sequence, which represents the up to 2%–3% of the human genome in which variants frequently have large phenotypic effects (Adzhubei et al. 2010; Sim et al. 2012; Ritchie et al. 2014; Hecht et al. 2015; Rentzsch et al. 2019). In fact, the current ACMG guidelines only allow the clinical classification of coding sequences, which represent only ∼1% of the genome (i.e., transcript exon portions translated to amino acids; Fig. 1B). However, generally exonic sequences including noncoding exons like 3′ and 5′ untranslated regions (UTRs) or sometimes also retained introns have been studied long before the availability of a human reference genome from so-called complementary DNA (cDNA) libraries and expressed sequence tags (ESTs). With a transition to genome capture approaches and ES, we can see exon proximal sequences in our analyses, like a few intronic bases or (parts of) the transcript promoter. Although we see a clear enrichment of coding variants in databases like ClinVar (Fig. 1B), the majority of variants associated with common diseases, as well as an unknown proportion of causal variants for rare diseases, fall into the remaining “noncoding” regions of the genome (Chatterjee and Ahituv 2017).

This includes a wide set of potential molecular processes like a diverse group of short and long RNA species, untranslated sequences of all transcripts (e.g., 3′ UTRs, 5′ UTRs, introns including proximal, and distal splice recognition sites or circular RNAs) as well as repeats and satellite sequences. Further, various regulatory sequences are a subset of the noncoding space. This jointly refers to sequence changes in promoter and distal regulatory elements like enhancers, repressors, or insulators including so-called topologically associated domain (TAD) boundaries (Gasperini et al. 2020). One important aspect of gene regulation by these regulatory elements appears to be related to the 3D architecture of the genome in the nucleus and had been largely ignored as a disease mechanism in the past (Spielmann et al. 2018). With the discovery of TADs and our increased knowledge about regulatory elements and DNA folding, we are now able to consider the positional effects and regulatory-element adoption for their role in human disease (Lupiáñez et al. 2015; Franke et al. 2016; Flöttmann et al. 2018; Elsner et al. 2021; Socha et al. 2021). Regulatory sequences cover 5%–20% of the genome (e.g., ∼18% in the annotation used in Fig. 1B) and are supposedly highly enriched for the remainder of the undiscovered disease-causing and functional variants. Although known phenotypic effects of regulatory variants are more subtle than those of coding changes, they are thought to, for example, underlie most of the known primate species differences (King and Wilson 1975) and large proportions of the phenotypic variation among humans (Stranger et al. 2007; Albert and Kruglyak 2015; Blake et al. 2020).

As mentioned above, a starting transition from panels or exomes to “whole” genomes (genome sequencing, GS) enabled by a reduction in sequencing costs has not substantially increased diagnostic rates. GS has rather been used to improve data quality and the ability to call structural and copy-number variation due to a more even sequence coverage (Kingsmore et al. 2019; Lowther et al. 2020; Brockman et al. 2021). Further, like with the initial transition from panels to exomes, the GS data is frequently computationally restricted to an exome or even a panel equivalent. Reasons for that are manifold and range from computational reasons (i.e., reducing processing times), to legal and reporting considerations (e.g., preventing incidental findings), over to a perception that the number of variants and their diverse potential molecular effects is not manageable. The result is a hierarchical approach in which tiers of analysis are performed depending on whether a plausible variant was identified from the earlier tier. This inherently creates a confirmation bias and reduces the chances of finding noncoding or polygenic causes of disease. In line with these considerations, we have not seen a substantial increase of noncoding pathogenic variants in ClinVar over the last years (Fig. 1B).

## COMPUTATIONAL PREDICTORS OF VARIANT EFFECTS

Variant function or molecular effect may be obtained from experimental studies or can be the result of computational predictions. Variant catalogs, as discussed below, may serve as lookup tables for variant function, but typically our knowledge of individual variants is still far from comprehensive, especially considering the vast universe of potential sequence alterations that can be created. Therefore, computational models and algorithms that predict functional consequences of variants are often the basis of an informed clinical assessment. However, according to the current ACMG guidelines, computational evidence is set to be only “supporting evidence.” Various approaches and tools are used to screen and prioritize large numbers of variants (McLaren et al. 2010, 2016; Wang et al. 2010; Paila et al. 2013; Flygare et al. 2018), providing a relative ranking of potentially causal variants for further follow-up. Some of the resulting computational scores have been used in the field for more than a decade (Ng and Henikoff 2003; Adzhubei et al. 2013), and scores like the Grantham score of missense variants date even further back (Grantham 1974). Generally, there is a large number of scores developed to prioritize missense variants (Hecht et al. 2015; Ioannidis et al. 2016; Sundaram et al. 2018; Livesey and Marsh 2020; Pejaver et al. 2020; Reeb et al. 2020), but other specialized scores (e.g., for synonymous [Buske et al. 2013; Zeng and Bromberg 2019] or splicing variants [Jian et al. 2014; Rosenberg et al. 2015; Cheng et al. 2019; Riepe et al. 2021]) are also available.

### The Power of Specialized Scores

It seems useful to distinguish scores that are used for a specific (molecular) function (like those for missense, synonymous, and splice effects, but also specialized predictions of protein phosphorylation sites, transcription factor, or miRNA binding) from those that are broadly applicable (like conservation or variant density derived metrics). Currently, the vast majority of available computational predictors or scores are “specific.” Especially when predictors are trained from experimental or otherwise curated data, the resulting predictor is typically limited to the domain that its training data was derived from and performing very well in this specific domain. However, related to the training data, any kinds of ascertainment issues have severe consequences for the resulting model that are frequently not considered by users applying these tools. For example, in curated databases, genes with higher evolutionary conservation might be overrepresented because of a historically earlier description in the scientific literature. This will propagate into models as an increased weight of sequence conservation. Similarly, biases occur because an experiment is unable to measure some kinds of variant effects; for example, in splicing when only a limited sequence context around the splice donor or acceptor is measured, the model will have no power for intronic splicing factors (Rosenberg et al. 2015). In this context, it is also important to point out that a variant can have effects on multiple molecular functions like changing an amino acid as well as splicing and that specialized models are not correctly capturing these effects (Rentzsch et al. 2021).

There are different areas (e.g., various RNA species like long noncoding or RNAs or miRNAs and their genomic targets, transcript stability, repeat elements, genomic architecture) where computational effect predictions still need substantial improvement. These areas typically correspond to molecular functions that are mechanistically not yet completely understood or for which only experiments with limited throughput exist. The largest class with limited computational effect prediction by genomic sequence are regulatory sequences. When correlating regulatory effects in experimental data with multiple integrative scores combining sequence conservation, functional element annotations, in silico transcription factor (TF) binding site predictions, or biochemical readouts (e.g., TF immunoprecipitation, histone mark immunoprecipitation, or open chromatin signals), we previously found that no score or annotation consistently predicts the results (Inoue et al. 2017; Kircher et al. 2019). Existing gene regulatory scores excessively rely on conservation and are mostly unable to predict gains of TF binding. Sequence-based models (e.g., using gapped-string kernels [Ghandi et al. 2014; Lee et al. 2015] or convolutional neural networks [Avsec et al. 2021a,b; Ching et al. 2018]) overcome some of these limitations and show the overall best performance (Shigaki et al. 2019). However, the development of improved predictors of regulatory sequence effects will remain a very active field of research for the next years.

Another field that has seen advances from the application of deep-learning models are protein structures. A recent publication on unsupervised models of missense effects based on protein structure highlighted the potential of neural networks (Frazer et al. 2021), but also the high variance across proteins and the challenges of covering all proteins. In regards to a comprehensive coverage, the Alphafold2 model (Jumper et al. 2021) has recently received a lot of attention for the inference of protein structures from only sequence. Inferring protein folding can be instrumental for understanding amino acid impact (e.g., due to identification of interacting residues) and may therefore provide important information in missense classification. We will likely see a number of tools that use this data over the next year. However, many molecular functions are not directly inferred from structure (Li et al. 2010; Vacic and Iakoucheva 2012; Reimand et al. 2015; Sahni et al. 2015; Lugo-Martinez et al. 2016). For example, a protein might lose or gain phosphorylation sites critical for its function and we would not necessarily see a change in folding. Similarly, changes in a potential binding pocket might not affect the major ligand binding or even enable binding of additional ligands. Therefore, it remains unclear whether these advances in predicting protein structure will also translate in significantly better prediction of pathogenic amino acid exchanges (Diwan et al. 2021), especially given the very good existing performance.

In this context, it should also be mentioned that existing missense scores, especially those that highly correlate with evolutionary conservation, do not always correlate well with the results of deep mutational scanning (DMS) screens as discussed below (Gray et al. 2018; Livesey and Marsh 2020; Reeb et al. 2020). To this point, it is unclear whether this is related to the limitation of these screens testing specific protein characteristics (e.g., stability and folding) or functions (e.g., survival, abundance, binding, metabolic products) or whether available missense scores were inherently biased in their development by using conservation or the representation of certain protein classes (e.g., globular, highly structured).

### Combined and Universally Applicable Scores

Given a broad and unbiased data set, it is possible to use machine learning to integrate various annotations and specific scores to a broadly applicable metric. Tools like Eigen (Ionita-Laza et al. 2016), LINSIGHT (Huang et al. 2017), or CADD (Kircher et al. 2014) are applying such strategies. The combined metrics are very convenient for users as they make use of many different annotations that are all partially correlated (and could not be considered independent evidence) and also allow to assess variants of potentially different molecular function (e.g., coding vs. splicing vs. regulatory) on the same numerical scale. The limitations of such approaches are again with the ascertainment of the training data set as well as the coverage of the measures that are being integrated. For example, if there are no features that cover regulatory effects, the model will not be predictive for that. Similar limitations apply if certain functional classes are not well-represented in the training examples.

Even though widely adopted (Adzhubei et al. 2013; Carter et al. 2013; Dong et al. 2015; Ioannidis et al. 2016; Jagadeesh et al. 2016; Pejaver et al. 2020), the general approach of using available clinical variant data sets to train models or integrate data for the prediction of pathogenicity needs to be strongly cautioned. Although many of the published methods implement theoretical and practical measures to assess their ability to generalize, if data limitations are not appropriately corrected, the resulting models still suffer from ascertainment bias and circularities in the variant interpretation process. For example, variants in clinical databases cluster around well-described disease genes—that is, the number of years that a gene has been associated with a disease will affect the number of reported variants. This number also correlates with the gene's species conservation and when its cDNA was described for the first time. Further, certain diseases (and biological functions) have been getting more attention over the years (e.g., brain, heart, limbs), causing a representation bias. In addition, certain genes or proteins allow easier experimental follow-ups (e.g., metabolic enzymes vs. membrane proteins). There is also an enrichment for high impact effects on the variant level (see also Fig. 1B) and for variant location within the genes—for example, variants in binding pockets are enriched over variants positioned in less-constrained protein-interaction domains. As a result, variants reported to clinical databases tend to have high conservation scores, low population frequency or are absent from data sets, are located away from repeat rich sequence, are discovered for “more common” rare diseases (because it is easier to recruit patients), or are identified in inbred populations. These are just a few criteria and they are to some extent directly manifested in the guidelines of the ACMG and others (Richards et al. 2015). This applies not just for pathogenic variants, but a considerable proportion of the reported benign variants may have been considered a plausible candidate for a pathogenic variant and have subsequently been excluded (partially based on ACMG criteria and not necessarily experimental results).

The development of various computational tools, pipelines, and predictive models requires a transparent and rigorous benchmarking and validation process. In this context, several editions of the Critical Assessment of Genome Interpretation (CAGI) challenges have contributed by bringing various computational developers and real-world data producers together (Andreoletti et al. 2019). Challenges typically include two parts: a data set on which the developers can directly evaluate and maybe even adjust their methods and a second data set for which the correct answers are only revealed after the conclusion of the challenge. In contrast to performance evaluations commonly presented for tools at time of publication, overfitting to the limited amount of validation data is prevented. CAGI challenges are diverse and, for example, ask submitters to either score specific molecular functions (e.g., amino acid exchanges, splice sites, regulatory sequences) or benchmark whole pipelines, like nominating disease causal variants from whole-genome sequencing (with or without knowledge of the disease phenotype). The continued effort of developing highly sensitive specialized scores for different molecular functions and their subsequent integration in a broadly applicable metric will be the foundation for a better prioritization of all genomic variants and will make sure that the interaction of several molecular function at a certain genomic site is considered (Rentzsch et al. 2021).

Another important distinction between different computational methods is the range of variants that can be scored. For example, many tools are limited to SNVs and cannot handle multinucleotide variants, indels, or SVs (defined as insertions, deletions, inversions, or translocations of >50 bp). Although still not widely implemented, scoring of indel changes has gained attention over the last years (Kircher et al. 2014; Folkman et al. 2015; Douville et al. 2016; Pagel et al. 2019). During recent years, structural variants have seen technological and algorithmic advances, improving the quality and number of events that are being identified (Sudmant et al. 2015; Ebert et al. 2021). Despite SVs being the smallest class in absolute numbers (typically fewer than 20,000 identified per individual), the number of nucleotides affected typically exceeds those of the other variant classes combined. SVs are therefore a prominent variant class when it comes to increasing the diagnostic yield. From a computational perspective, they are challenging as annotations need to be aggregated across large genomic regions and the potential molecular effects at play may be diverse (Ganel et al. 2017; Geoffroy et al. 2018; Kumar et al. 2020; Kleinert and Kircher 2022; Sharo et al. 2022). Specifically, effects might be due to dosage effects or due to regulatory changes in the local 3D architecture of the genome (Huynh and Hormozdiari 2019).

## EXPERIMENTAL ASSESSMENT OF VARIANT EFFECTS

As outlined above, the widespread introduction of next-generation sequencing (NGS) technologies and GS in the clinical routine has led to a massive increase in the number of VUSs. Except for obviously pathogenic nonsense and canonical splice site variants, one of the most common wetlab-based methods for testing the pathogenicity of a variant is family segregation analysis. Additionally, the detection of a de novo mutation is clinically considered a good indication of the pathogenicity of a variant (Veltman and Brunner 2012). The Deciphering Developmental Disorders study and the U.K. 100,000-genomes project showed that ∼40% of all patients with developmental delay carry a pathogenic de novo mutation in their coding sequence (Short et al. 2018; 100,000 Genomes Project Pilot Investigators et al. 2021). Functional follow-up assays are then applied to each VUS as they are encountered in patients. Although this de novo approach might be feasible for panels and ES, the introduction of GS increases the number of de novo variants to 70–100 per trio and makes it experimentally impossible to assess which variant is pathogenic (Turner et al. 2017).

### Multiplex Assays of Variant Effects (MAVEs)

Therefore, there is an urgent need to develop “next-generation functional tests” for the comprehensive and systematic evaluation of thousands of variants from GS data. Multiplex assays of variant effects (MAVEs), which encompass strategies for coding and noncoding sequences, can be used to overcome this shortage (Inoue and Ahituv 2015; Starita et al. 2017). Several recent articles review the technical aspect of MAVEs (Inoue and Ahituv 2015; Starita et al. 2017; Findlay 2021) and a comprehensive repository is available online (https://www.mavedb.org) with MaveDB (Esposito et al. 2019).

**Figure 2.**
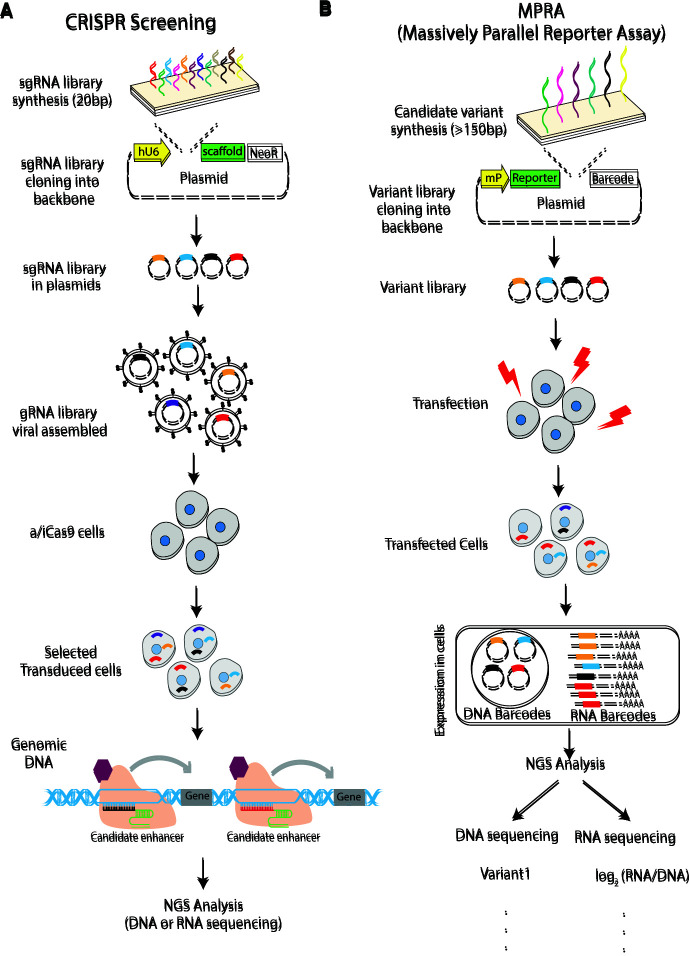
Multiplex assays of variant effects (MAVEs): Clustered regularly interspersed short palindromic repeat (CRISPR)-based (*A*) and massively parallel reporter assay (MPRA)-based (*B*) MAVE strategies share a common framework. First, hundreds or thousands of genetic variants are created (e.g., by synthesis or error-prone polymerase chain reaction) and cloned into a plasmid system. Second, this mutant library is introduced into an in vitro system and finally read out by a biological phenotype or function using massively paralleled sequencing. (sgRNA) Single-guide RNA, (gRNA) guide RNA, (NGS) next-generation sequencing.

The MAVE strategies developed for different applications all share a common framework (Fig. 2). First, hundreds or thousands of genetic variants are created (e.g., by synthesis or error-prone polymerase chain reaction) and cloned into a plasmid system. Second, this mutant library is introduced into an in vitro system and finally read out by a biological phenotype or function using massively paralleled sequencing (Starita et al. 2017). This high-throughput approach in principle allows for systematic screening of all possible nucleotide variants within a gene or region of interest. What makes MAVEs highly scalable is that variants are engineered and tested in a pooled format, drastically reducing cost and minimizing sample processing (Findlay 2021). Here we will briefly introduce the different approaches and then discuss how they could be translated into the clinic.

### Coding Variants: Deep Mutational Scanning

Fowler et al. (2010) introduced the concept of deep mutational scanning to study the effect of amino acid substitutions on protein function by profiling the protein binding properties of more than 600,000 variants of the human WW domain. The authors presented a high-resolution map of mutational effects across the WW domain and could show that each position had unique features that would have taken many years to capture by identifying a few representative mutations. Since then, this approach has successfully been applied to several disease loci (Harris et al. 2016; Koenig et al. 2017; Mighell et al. 2018, 2020; Schmiedel and Lehner 2019; Esposito et al. 2019; Chiasson et al. 2020; Starr et al. 2020; Sun et al. 2020; McCormick et al. 2021).

The widespread introduction of CRISPR–Cas9 paved the way for the development of in-genome MAVEs that consider the local genomic context. Findlay et al. (2014) coupled CRISPR–Cas9 genome editing with multiplex homology-directed repair using a library of donor templates carrying all possible SNVs in exon 18 of *BRCA1*. As phenotypic readout the authors measured variant effects on nonsense-mediated decay, exonic splicing, and cellular growth. This saturation genome editing approach was later expanded to 13 critical exons of *BRCA1* covering 96.5% of all possible SNVs (Findlay et al. 2018). Of the almost 4000 experimentally tested variants, 25% of VUSs and 49% of variants with conflicting previous reports could be flagged as nonfunctional. It was estimated that saturation genome editing has >95% accuracy in predicting the functional outcome of a genetic variant in *BRCA1*.

### Noncoding Variants: MPRAs/CRE-seq and STARR-seq

The identification and interpretation of medically relevant noncoding variants represents a major bottleneck in human genetics. Massively parallel reporter assays (MPRAs, sometimes also referred to as CRE-seq or STARR-seq) enable thousands of regulatory elements or mutated regulatory elements to be concurrently assayed in a single, quantitative experiment (Kwasnieski et al. 2012, 2014; Melnikov et al. 2012; Arnold et al. 2013, 2014; Kheradpour et al. 2013; White et al. 2013; Birnbaum et al. 2014; Shlyueva et al. 2014; Inoue and Ahituv 2015; Muerdter et al. 2015; Klein et al. 2020). This is achieved by synthesizing libraries of potential regulatory elements including unique molecular barcodes that can be analyzed by high-throughput sequencing. In the case of STARR-seq assays (Arnold et al. 2013, 2014; Muerdter et al. 2017), the insertion of regulatory element in the 3′ UTR serves as the molecular barcode directly. A limitation of the early implementations of the MPRA method was that the assayed DNA is episomal and does not integrate into the genome. In contrast, lentivirus-based MPRAs (Inoue et al. 2017; Gordon et al. 2020) enable genomic integration of assayed sequences, including the ability to infect difficult to transfect cells, such as neurons (Inoue et al. 2019). To minimize positional effects of random integration, flanking insulators to the vector (pLS) are also included.

By measuring allelic pairs, or allelic series up to comprehensive saturation mutagenesis libraries, individual variant effects can be inferred from MPRAs. Large MPRA data sets, measuring regulatory variant effects, are currently limited to specific loci (Kircher et al. 2019), derived from readouts of only standing variation (e.g., quantitative trait locus [QTL] studies, common variants, or variants identified across cancer cell types [Tewhey et al. 2016; van Arensbergen et al. 2019]) or are only available for single cell types (Tewhey et al. 2016; Kircher et al. 2019). Generally, MPRAs are still mostly used to measure the activity of regions, not the effect of individual variants.

CRISPR–Cas9 genome editing has also influenced the way that the noncoding genome is currently being investigated. CRISPR screening experiments in combination with state-of-the-art single-cell technologies now enable mapping of noncoding elements in thousands of different loci in a single multiplexed experiment (Gasperini et al. 2019). The most common types of CRISPR screening modalities have recently been reviewed by Przybyla and Gilbert (2021).

### Inherent Limitations and Biases of MAVEs

One of the key limitations of all MAVEs in the context of developmental disorders and rare disease is the lack of appropriate tissues or cells that are needed to perform a high-throughput functional assay. Although immortalized cell lines can easily be transfected with complex libraries, they do not resemble the aspects of embryonic development. Further, studies have shown that the results of, for example, MPRAs are dependent on the cell type that is used (Maricque et al. 2017; Inoue et al. 2019; Kircher et al. 2019; Griesemer et al. 2021). For deep mutational scanning, sequence length and complexity restrictions also limit which proteins can be assayed.

The noncoding genome represents yet again a particular challenge: Because of current restrictions in DNA synthesis, the fragments that are assayed in an MPRA are usually between 150 and 300 bp in size. However, there are many examples of enhancers and regulatory landscapes that are longer than that. These long regulatory elements are thought to drive tissue specific gene expression through chromatin folding in the 3D architecture of the nucleus. These are aspects that are currently not considered with MPRAs.

Another bottleneck is the fact that even when applying MPRAs, the final validation experiment of whether a DNA sequence is an enhancer and whether a variant alters it can currently only be done by an in vivo (reporter) assay. This is usually performed in zebrafish or in transgenic mice (Visel et al. 2009; Franke et al. 2016). The DNA sequences are cloned into a reporter construct, which usually consists of a minimal promoter and LacZ or green fluorescent protein (GFP). This construct is then tested by injection in a model organism. An enhancer then leads to tissue-specific expression of the reporter gene. For example, an enhancer of the extremities shows a specific staining in the extremities of the mouse embryo. The entire procedure can take up to several months and is limited in throughput.

A final limitation is the fact that MPRAs and CRISPR-based assays are technically very demanding. Routine laboratories will probably not perform MAVEs in a clinical setting. It seems more likely that highly specialized academic centers could perform these analyses for a particular region of the genome or a disease of interest, providing the resulting data to the medical community. National and international funding will be necessary to perform these experiments at scale and to organize access to the resulting data.

## CATALOGS OF VARIANT EFFECTS

In addition to variants identified from population genetics studies, an increasing number of samples from various clinical studies have been aggregated and used to catalog known genetic variation. Samples collected as controls in disease studies or for population studies are obtained from healthy individuals. This never excluded the possibility of late onset disease variants in the resulting databases, but in recent years, individuals with known diseases were also actively included in variant databases, if their phenotype was not severe or defined as late onset. As a result, variant frequency always needs to be interpreted for the specific disease and variant, and no conclusion can be drawn from the mere presence or absence. To this point, it is also important to note that the majority of currently known variants are singletons, that is, variants identified from one individuals’ genome. Despite what seems a shallow sampling (around 100,000 individuals from a population of billions) of the overall variation that is compatible with life, combining this information as variant density (i.e., the underrepresentation of common variants or the clustering of disease-associated variants in a region) may be used as evidence for prioritizing functional variants (di Iulio et al. 2018; Havrilla et al. 2019). Databases of known variants like gnomAD (Karczewski et al. 2020) or BRAVO (Taliun et al. 2021) are therefore an important source.

Similarly, collections of potential disease causing variants are highly relevant. This obviously includes collections like ClinVar (Landrum and Kattman 2018) or variants curated from literature like HGMD (Stenson et al. 2020), but also variants implicated by genome-wide association studies (GWASs) and QTL studies like the GWAS catalog (Buniello et al. 2019) or the GTEx expression QTLs (GTEx Consortium 2020). There is also a huge value in making information about variants that are being considered as candidates for certain diseases or phenotypes available. In the most basic sense, these are the VUSs that we find in ClinVar, but should also include unpublished analyses and the options to collaborate directly with other researchers in establishing the disease link through platforms like MatchMaker Exchange (Philippakis et al. 2015).

Another rich and underused source of information is the growing amount of molecular data that is available for human, but also for model organisms (Wangler et al. 2017; Shefchek et al. 2020). On the one hand, there are the results of assays as we discussed them here and that are, for example, made available through MaveDB (Esposito et al. 2019). On the other hand, there are tens of thousands of functional genomics data sets available, for example, through the NBCI Gene Expression Omnibus (Barrett et al. 2013)—including gene expression, immunoprecipitation of DNA binding (TFs and histones), DNA accessibility, DNA methylation, 3D organization, and interaction of DNA elements available for various cell types, whole tissues, or single-cell experiments. To give an example of how such data can be used for the identification of mutations in enhancers and functional elements, variant positions can be overlaid with histone marks or the VISTA database (Visel et al. 2009; Spielmann et al. 2018).

The available functional genomics data is vast and highly valuable. Still, only a minority of variants in patients with developmental delay lie in known enhancer elements characterized by histone marks (Short et al. 2018; Moore et al. 2020). Furthermore, regulatory gain of function mutations (i.e., variants that generate new transcription factor binding sites) might not be recognized by this approach. Another general challenge is that these data sets are created and analyzed by many different laboratories, making it difficult to identify the most relevant data sets and to compare or jointly analyze different data sets. In this context, it seems important to mention the ENCODE and NIH Roadmap Epigenomics Mapping consortia (The ENCODE Project Consortium 2012; Roadmap Epigenomics Consortium et al. 2015) again, as they have initiated data portals with versioned and uniform data processing pipelines as well as explored the possibility of imputing the results of certain molecular assays from other assay data. They are also the data source for several efforts to annotate functional elements and regions across available cell types, like the SCREEN database (Moore et al. 2020) or ChromHMM segmentations (Ernst and Kellis 2017; Vu and Ernst 2022).

The increasing number of experimental element and variant readouts, a huge library of functional genomics tracks and annotations, and a “zoo” of computational models (Avsec et al. 2019) and tools is very challenging to navigate and currently impossible to integrate into variant analysis for single laboratories or even larger institutions. What is clearly required is a one-stop solution, a website and database where currently available information about a genomic alteration is aggregated. One initiative in this space is the above-mentioned IGVF Consortium, one of the successor efforts of the highly visible ENCODE consortium. In addition to using state-of-the art experimental as well as modeling approaches to create more data, the IGVF consortium also aims to build a variant effect catalog as a resource for the broader research community that catalogs variant impacts including the underlying data, tools, and models (National Human Genome Research Institute (NHGRI) 2021). For this goal, the consortium includes two Data and Administrative Coordinating Center awards that will support this process. We believe it is critical to integrate this into a larger effort of data coordination centers also in other national initiatives and to allocate resources to make the available information accessible, as a service to the genetics community and most importantly also the patients.

## CONCLUSIONS AND FUTURE DIRECTIONS

The broad introduction of NGS technologies into patient care has revolutionized human genetics. Although currently the major focus still lies in the in-depth diagnostics of rare diseases, consultation of “critically ill” infants, and complex syndromic cases, the development of a precision medicine is on our horizon. Over the next years, the focus will expand toward common disease, precision oncology, and eventually even the treatment of genetic disorders. However, the foundation of this path toward genomic medicine will be through careful variant interpretation. The number of VUSs will continue to rise. Despite major advances in the computational and experimental methods that we described here, it will remain unfeasible to test large numbers of variants with multiple assays or in a large number of biological conditions. Without further knowledge and more experimentally validated noncoding variants, the medical interpretation of variants in noncoding DNA will remain one of our biggest challenges. Other major challenges are polygenic and oligogenic variants. At least for the next few years, we will struggle with a good balance of what variants to report to the patients and how to implement effective cycles of variant reinterpretation.

We have outlined the clinical need for improved data integration, summarization, and presentation. One approach that we start to see, and that might be very promising in reducing the sheer number of individual data sets, is replacing a large number of functional genomics data by its representation learned in convolutional neural networks. Here, sequence regions (potentially of several kilobases of sequence) are used as the input for predicting molecular readouts like open chromatin regions, histone marks, or DNA methylation (Zhou and Troyanskaya 2015; Kelley et al. 2016; Zhou et al. 2019; Schwessinger et al. 2020; Avsec et al. 2021a,b). This potentially removes biases by combining many experiments, generalizes sample-specific information (potentially easing model sharing even if data cannot be shared), and allows the prediction of the molecular data for new DNA sequences. Especially the application to new DNA sequences will be key to predicting allele-specific effects and to performing in silico mutagenesis studies. The growing efforts of establishing cell atlases and developmental trajectories from single-cell data (Cao et al. 2020; Haniffa et al. 2021) will hopefully help in connecting what are currently separate cell-type or tissue readouts to a continuous trajectory of the specific molecular function.

Variant prioritization profits from the specialized models of certain molecular processes (including sequence- and deep-learning-derived models) and their subsequent integration across various potential molecular effects to genome-wide scores. The next generation of these integrated scores needs to be able to score all kinds of variant types from SNVs over multinucleotide changes to chromosomal alterations. They also should be developed in a way that makes use of cell-type-specific effects while weighting such contributions in an organismal score. Consequently, our ability to predict and experimentally assess the effects of genetic variants will undoubtedly continue to improve, but it will be imperfect as long as it is based on several layers of approximations of molecular processes.

The goal should be a comprehensive computational support for clinical genetics. Ideally, this would be possible with a single tool that clinicians could use when diagnosing their patients. Although this seems not possible right now, we start by data integration in high-level frameworks for variant interpretation that use as much available information as possible. This includes various types of information like segregation, allele frequency, affected cell types and tissues, gene expression, molecular pathways, computational effect predictions, phenotypic effect, and (deep) phenotyping data. This information would be considered in an integrated likelihood or multiple-hypothesis-testing framework. Such framework could be seen as extension or generalization of what is currently done when considering multiple disease models (e.g., recessive, compound heterozygote, dominant, de novo, or mosaicism) in analysis. As pointed out earlier, the number of variants identified in an individual genome is too large for considering any unconstrained combination of potentially causal variant alleles. Instead, information on the phenotype might nominate relevant cell types and pathways, thereby prioritizing potential gene sets and regulatory regions for which polygenic variant sets could be considered. The complexity of the considered hypotheses might be scaled by omnigenic genetic burden estimates (i.e., risk scores) used as proxies of how much buffering or compensation might be masking the severity of the phenotype in the genetic background of the patient. We might, for example, expect high impact and monogenic cause, if the patient does not have a high genetic burden, whereas in the case of a high genetic burden, complex interactions of subtle effects might be disease causal. Most importantly, such an analysis should not be performed in tiers, but as a ranked list in which hypotheses are invalidated by additional evidence like functional or genotypic data. To this end, systematic and objective clinical guidelines will need to evolve with active involvement from computational method developers, and clinicians, counselors, and eventually patients will have to embrace a more quantitative integration of evidence rather than the strict classification (Tavtigian et al. 2018, 2020).

Our goal is the universal and fully integrated software for variant interpretation. However, we know that this is currently unreasonable. This would require high levels of standardization and FAIR (findability, accessibility, interoperability, and reusability) data principles that the field just starts to address. Another challenge is the “*n* + 1” problem—that is, what to do when additional data of one more sample or one more experiment needs to be integrated in the models. This creates version cycles and requires revisiting all previous results after such updates. To justify the computationally expensive process of retraining and reanalysis, this is only reasonable when adding a substantial amount of new data. Further, it seems important to stress that results of any ranking of potentially causal variants will need to be transparent. We should aim for a common reporting standard that makes it possible to understand why a certain variant set is suggested as causal and what the major underlying processes are. This information needs to be at such a level and of such clarity that it can be validated and also be provided back to the patient for informing future medical treatments.

## ADDITIONAL INFORMATION

### Acknowledgments

We thank current and previous members of the Kircher and Spielmann laboratories for helpful discussions and suggestions. We thank Verónica Yumiceba Corral for her help with Figure 2. We thank three reviewers for their comments and feedback.

### Funding

M.S. is supported by grants from the Deutsche Forschungsgemeinschaft (DFG) (SP1532/3-1, SP1532/4-1, and SP1532/5-1), the Max Planck Society, and the Deutsches Zentrum für Luft- und Raumfahrt (DLR 01GM1925). M.K. is supported by the NIH/NHGRI IGVF effort (1UM1HG011966-01).

### Competing Interest Statement

The authors have declared no competing interest.

### Referees

Elizabeth J. Radford

Anonymous
